# The impact of socio-demographic features on anxiety and depression amongst navy veterans after retirement: a cross-sectional study

**DOI:** 10.1186/s13104-020-04966-x

**Published:** 2020-03-03

**Authors:** Dimitris Georgantas, Andreas Tsounis, Ioannis Vidakis, Maria Malliarou, Pavlos Sarafis

**Affiliations:** 1 Hellenic Open University, Patra, Greece; 2 School of Psychology, Aristotle University of Thessaloniki, Thessaloniki, Greece; 3 Faculty of Nursing, University of Thessaly, Larissa, Greece; 4 Department of Nursing, School of Health Sciences, Cyprus University of Technology, 15, Vragadinou Str, 3041 Limassol, Cyprus

**Keywords:** Anxiety, Depression, Navy, Veterans, Retirement

## Abstract

**Objective:**

Retirement from work may trigger various changes in everyday life that affect mental health. The current cross-sectional study, conducted with 231 veterans, examines the relationship between socio-demographic features and both anxiety and depression in navy veterans after retirement. Spielberg’s State-Trait Anxiety Inventory (STAI) was used for anxiety assessment, and the Beck Depression Inventory (BDI) was used for depression assessment. The analysis was performed with the Statistical Package for Social Sciences (SPSS), version 20.0.

**Results:**

It was found that the mean score of state anxiety was 41 and trait anxiety, 38. Severe depression was found in 6.5% of the veterans, moderate in 8.3% and mild in 21.7%. The presence of a serious health problems was an independent predictor of both anxiety and depression’s more serious symptoms. Inversely, the stability in terms of retirement choice was negatively related to depression, while the development of new interests and activities after retirement was negatively related to both anxiety and depression. Further, life satisfaction after retirement was a predictor of lower current anxiety levels among veterans.

## Introduction

Retirement is a major life transition that leads to various psycho-social changes. On the one hand, retirees may have more time to relax or spend time with family and friends, but on the other hand, due to role loss, they may undergo income reduction and be faced with boredom due to the absence of responsibilities [1]. Moreover, literature suggests that retirement may have both positive and negative effects on health [2]. Further, several studies support that retirement may lead to weight gain [3], increased smoking and alcohol consumption [4, 5], increased number of chronic conditions [6], decrease in cognitive function [7] and mental health deterioration [8]. Contrarily, according to other studies, retirement may result in better perceived physical [9], mental [10, 11] and general health [9, 12] as well as beneficial changes in health-related behaviors [11].

Nevertheless, the effects of retirement are related to many factors including socio-economic status, timing of retirement and type of job. According to the findings of a systematic review based on the results of 22 studies, retirement may lead to more positive mental and physical health effects among higher socio-economic groups [1]. Concerning its timing, research shows that early retirement might affect health, while continuing employment after the expected retirement age offers no health benefits, indicating that retiring around the expected age is optimal [13, 14]. Additionally, mandatory retirement may lead to more negative health and social effects as compared to voluntary retirement [15]. As far as the type of work is concerned, in the case of jobs with hazardous exposure, higher stress and excessive physical strain, exiting the workforce early may be beneficial for health [2]. However, retirees from labor positions that involve physical strain may experience weight gain due to the subsequent reduction in physical activity [16].

Although challenges related to the retirement transition period are common for most people, retirement may prove even more difficult for army veterans as the transition from a professional environment with strict rules, strong hierarchy conditions and varied social networks may make it more difficult to adapt to a new life. Additionally, army veterans endure the transition from frequent transfers to different units and cities to a stable home [17].

Thus, the aim of the current study is to examine the impact of socio-demographic characteristics on anxiety and depression in navy veterans after retirement. To our knowledge, no other study has been conducted involving navy veterans in Greece, and the majority of studies concerning veterans’ retirement is not targeted to a specific population.

## Main text

### Methods

#### Participants and procedure

The study was conducted between November 2016 and April 2017 for which self-administered questionnaires in closed envelopes were distributed to 600 navy veterans by the researchers at various places that are potentially frequented by veterans such as the National Union of Navy Retirees, the Cultural Association of Veterans and Athens Naval Hospital. In most cases, the participants returned the closed envelopes to competent members of the unions or employees of the hospital who had been designated as reference persons by the researchers; however, in some cases (about 20%), the closed envelopes were directly returned to the researchers. Participants were informed about the purpose, procedure of the research and the fact that they could decline to participate at any time during the procedure. They were also informed that their participation was interpreted as informed consent and were reassured that all data was kept confidential. The final sample consisted of 231 veterans (response rate: 38.5%).

### Measures

#### Socio-demographics, retirement procedures and life after retirement

The research tool contained 17 questions recording both socio-demographic features and additional information concerning the retirement procedure and life after retirement including: gender, age, family status, Body Mass Index (BMI), the presence of a serious health problem, years of service, age of retirement, years of retirement, stability in retirement choice, desire to return to the navy, whether it was forced or voluntary retirement, smoking and alcohol consumption, satisfaction in personal career, preparation for retirement, development of interests and personal activities and life satisfaction after retirement.

#### Spielberg’s State-Trait Anxiety Inventory (STAI)

The STAI was used to measure anxiety [18]. It is a 40-item scale comprising two 20-item sub-scales. The state sub-scale evaluates the current state of anxiety, while the trait sub-scale refers to general tendency of individuals responding with anxiety to perceived environmental threats [18]. Form X of the STAI, which was used in the current study, has been translated and validated in Greek [19] and has been widely used on the Greek population.

#### Beck Depression Inventory (BDI)

The BDI was used for depression assessment [20]. It comprises 21 questions ranked on a 4-point scale (0–3). Cut-off scores are as follows: (i) 0–9 minimal depression, (ii) 10–18 mild depression, (iii) 19–29 moderate depression and (iv) 30–63 severe depression. The BDI has been translated and validated in the Greek language [21] and has also been widely used on the Greek population.

#### Statistical analysis

Multivariate Linear Regression analysis with the stepwise method was used to detect independent factors associated with anxiety and depression. Additionally, the analysis was performed using the SPSS, version 20.0.

### Results

The sample predominantly comprised males (93.1%) with the mean age of 59.9 years. Most participants were married (80.1%) with 50.6 years being the mean age of retirement and 9.8 years as the mean time from retirement. On the other hand, 51.1% retired on their own request, while 37% would return to the service if they could. Most participants were satisfied with their life after retirement (77.9%), although 46% of them were not prepared for it. The majority (86.6%) started new activities and developed new interests as veterans. Further, 23.4% were smokers, 9.5% reported systematic alcohol consumption and 14.3% had serious health problems. Moreover, income after retirement reduced for most of the veterans (81.4%), 21.6% worked although they retired, while 61% reported a desire for volunteering in the navy (Table 1).

**Table 1:** Socio-demographics and Retirement Issues (n = 231)

	Mean (SD)	N (%)
Gender		
Male		215 (93.1)
Female		16 (6.9)
Age	59.9 (8.1)	
Body Mass Index		
Normal		59 (25.5)
Overweight		100 (43.3)
Obese		72 (31.2)
Family status		
Married		185 (80.1)
Unmarried		12 (5.2)
Divorced		24 (10.4)
Widowed		10 (4.3)
Years of service	31.5 (4.6)	
Age of retirement	50.6 (4.7)	
Years in retirement	9.8 (8.7)	
Retirement after Veterans’ request		
Yes		118 (51.1)
No		113 (48.9)
Stability in retirement choice^a^		
Yes		100 (82.0)
No		22 (18.0)
Desire for return in the Navy		
Yes		85 (36.8)
No		145 (63.0)
Satisfaction from personal career		
Yes		186 (80.5)
No		43(18.6)
Life satisfaction as a retiree		
Yes		180 (77.9)
No		51 (22.1)
Being prepare for retirement		
Yes		124 (53.7)
No		107 (46.3)
Developing activities after retirement		
Yes		200 (86.6)
No		31 (13.4)
Smoker		
Yes		54 (23.4)
No		177 (76.6)
Systematic alcohol consumption		
Yes		22 (9.5)
No		209 (90.5)
Having a serious health problem		
Yes		33 (14.3)
No		198 (85.7)

^a^The question was addressed to those who answered yes in the previous question (n = 122)

Mean values, standard deviations (SD), potential range scores as well as the highest and lowest values of the scales and sub-scales of the study are presented in Table 2. The mean score of state anxiety was found to be 41 (SD = 11), trait anxiety was found to be 38 (SD = 11.2) and of depression was found to be 9 (SD = 7.6). Severe depression (according to the cut-off scores of BDI) was found in 6.5% of the veterans, while it was moderate in 8.3%, mild in 21.7%, and 63.5% did not display depression symptoms.

**Table 2:** Descriptive Statistics of the Study Scales and Subscales (n = 231)

	Range	Lowest score	Highest score	Mean	SD
State Anxiety	20–80	23	71	41	11
Trait Anxiety	20–80	20	74	38	11.2
Depression	0–63	0	40	9	7.6

Further, multivariate linear regression analysis showed that BMI correlated independently with the trait anxiety sub-scale as those who were overweight had 3.35 times higher stress. Additionally, the presence of serious health problems was an independent predictor for both state (β = 7.43, p < .001) and trait anxieties (β = 8.50, p < .001) as well as depression (β = 9.98, p < .001). However, those who were happy with their choice to retire reported less depressive symptoms as compared to those who regretted it (β = − 5.37, p = .001). On the other hand, the desire to return to work was independently correlated with higher trait anxiety (β = 4.48, p < .001) (Table 3), and the development of new interests and activities after retirement was an independent predictor for both state (β = − 5.44, p = .003) and trait anxieties (β = − 6.12, p = .001) and depression (β = − 3.59, p = .037). Finally, those who were satisfied with their lives as retirees reported 7.79 times less state anxiety as compared to those who were dissatisfied (Table 3).

**Table 3:** Multivariate Linear Regression Analysis with step-wise Methods with state anxiety, trait anxiety and depression as dependent variables (n = 231)

	State anxiety		Trait anxiety		Depression	
	β^a^	SE^b^	β^a^	SE^b^	β^a^	SE^b^
Overweight (BMI)			3.35*	1.48		
Having a Serious Health Problem	7.43***	1.70	8.50***	1.82	9.98***	1.90
Stability in Retirement Choice					− 5.37**	1.55
Desire for Return in the Navy			4.48***	1.25		
Developing Activities after Retirement	− 5.44**	1.83	− 6.12**	1.84	− 3.59*	1.70
Life Satisfaction as a Retiree	− 7.79***	1.43				

*** p < .001**p < .01, *p < .05

^a^Regression Coefficient

^b^Standard Error of the Estimate

### Discussion

The current study explored the impact of socio-demographic features and issues concerning the retirement procedures and life after retirement on anxiety and depression levels among navy veterans after retirement. Our findings concerning depression (severe: 6.5%, moderate and mild: 30%) are comparable to those of a previous study conducted among 502 Greek military veterans that revealed 3.8% had severe depression, and 23.2% had depression symptoms that ranged from mild to moderate [17].

BMI was an independent anxiety predictor with overweight veterans reporting higher trait anxiety. Additionally, overweightness and obesity were found to be negatively related to mental health but positively related with symptoms of sleep disturbances [22] that may indirectly affect stress levels. Further, these findings are in agreement with other studies on veterans [17, 23, 24]. In this context, a study concerning the associations of anxiety and depression with BMI conducted among 177047 adults in US revealed that obese and overweight people were significantly more likely to be diagnosed with anxiety during their lifetimes [25].

Furthermore, health issues affect the levels of anxiety and depression in retirees. Findings showed that the presence of serious physical health problems was negatively related to anxiety and depressive symptoms. Moreover, a strong relationship between older adults’ poor physical health and anxiety and depressive symptoms is well-documented in the literature both for the veterans [26–30] and the general population [31–33]. Further, physical illness may increase the risk of anxiety and depression and vice versa, as people who experience mood disorders due to physical health problems tend to isolate themselves from others resulting in reduced social interactions that, in turn, affects acquiring health [34].

Our study results show that the development of new interests and activities is negatively related to both anxiety and depression. This is consistent with the findings of previous empirical studies that indicate that the advancement of new interests and challenges may contribute to the better adjustment of retirees in this new phase of life by enhancing their willingness to be productive and efficient to fulfill the new requirements, thus preventing the deterioration of their physical and mental health [35–38].

Meanwhile, life satisfaction after retirement proved to be a strong predictor of state anxiety with those being satisfied as retirees reporting lower trait anxiety as compared to those who were dissatisfied. Retirement is a major life transition with adverse effects. Literature suggests that the achievement of life satisfaction in the new phase of life is strongly connected with positive mental health status both in veterans [17] and other retirees [38].

While interpreting the above results, we must consider the fact that a low response rate may lead to non-response bias. Literature suggests that respondents in health surveys often report better health statuses than non-respondents [39–42]. However, according to other studies, low response rate does not necessarily indicate non-response bias [43, 44]. A study that assessed whether low response rates were associated with demographic representatives -in which 81 surveys with response rate varying from 5% to 54% were included—showed that the association was indeed positive but only very weakly [45].

However, in our study, non-responders and responders had similar characteristics. Moreover, low response rate may be partially explained by the following assumptions during the period of the study: the veterans were dealing with important personal matters due to Greek austerity and pension cuts that created overall feelings of negativity; they were possibly unaccustomed to participating in such research; some of them had no time to participate (when questionnaires were completed on the spot) as they may not have returned the questionnaires if they completed them another day.

## Limitations

- As the design of the study was cross-sectional, it constrained the inferences of causality, while the extent to which the results could be generalized was limited.
- Participants were selected on the basis of convenience.
- The fact that the Greek military personnel are generally unaccustomed to taking part in psycho-social research—especially on topics related to mental health—may have prevented some veterans from participating.
- The sample was not geographically representative.
- The response rate was low (38.5%). Despite the fact that non-responders and responders had similar characteristics, the prevalence of both anxiety and depression may be both underestimated and/or overestimated.

## Data Availability

A confidentiality agreement with participants prevents us from sharing the data.

## Abbreviations

BDI: Beck Depression Inventory
BMI: Body Mass Index
STAI: Spielberg’s State-Trait Anxiety Inventory
